# Prosthesis-Patient Mismatch Negatively Affects Outcomes after Mitral Valve Replacement: Meta-Analysis of 10,239 Patients

**DOI:** 10.21470/1678-9741-2019-0069

**Published:** 2019

**Authors:** Michel Pompeu Barros Oliveira Sá, Luiz Rafael Pereira Cavalcanti, Sérgio da Costa Rayol, Roberto Gouvea Silva Diniz, Alexandre Motta Menezes, Marie-Annick Clavel, Philippe Pibarot, Ricardo Carvalho Lima

**Affiliations:** 1 Department of Cardiovascular Surgery at the Pronto Socorro Cardiológico de Pernambuco (PROCAPE), Recife, PE, Brazil.; 2 Universidade de Pernambuco (UPE), Recife, PE, Brazil.; 3 Nucleus of Postgraduate Studies and Research in Health Sciences at Faculdade de Ciências Médicas and Instituto de Ciências Biológicas (FCM/ICB), Recife, PE, Brazil.; 4 Institut Universitaire de Cardiologie et de Pneumologie du Québec, Canada.

**Keywords:** Mitral Valve/Surgery, Heart Valve Prosthesis, Meta-Analysis, Prosthesis-Patient Mismatch

## Abstract

**Objective:**

This study sought to evaluate the impact of prosthesis-patient mismatch on the risk of perioperative and long-term mortality after mitral valve replacement.

**Methods:**

Databases were researched for studies published until December 2018. Main outcomes of interest were perioperative and 10-year mortality and echocardiographic parameters.

**Results:**

The research yielded 2,985 studies for inclusion. Of these, 16 articles were analyzed, and their data extracted. The total number of patients included was 10,239, who underwent mitral valve replacement. The incidence of prosthesis-patient mismatch after mitral valve replacement was 53.7% (5,499 with prosthesis-patient mismatch and 4,740 without prosthesis-patient mismatch). Perioperative (OR 1.519; 95%CI 1.194-1.931, *P*<0.001) and 10-year (OR 1.515; 95%CI 1.280-1.795, *P*<0.001) mortality was increased in patients with prosthesis-patient mismatch. Patients with prosthesis-patient mismatch after mitral valve replacement had higher systolic pulmonary artery pressure and transprosthethic gradient and lower indexed effective orifice area and left ventricle ejection fraction.

**Conclusion:**

Prosthesis-patient mismatch increases perioperative and long-term mortality. Prosthesis-patient mismatch is also associated with pulmonary hypertension and depressed left ventricle systolic function. The findings of this study support the implementation of surgical strategies to prevent prosthesis-patient mismatch in order to decrease mortality rates.

**Table:** 

Abbreviations, acronyms & symbols	
CI	= Confidence interval
iEOA	= Indexed effective orifice area
LVEF	= Left ventricle ejection fraction
MVR	= Mitral valve replacement
OR	= *Odds Ratio*
PPM	= Prosthesis-patient mismatch
PRISMA	= Preferred Reporting Items for Systematic Reviews and Meta-Analyses

## INTRODUCTION

### Rationale

Recent meta-analyses including several studies with thousands of patients have been published in order to evaluate whether prosthesis-patient mismatch (PPM) is a risk factor for short- and long-term mortality after aortic valve replacement, showing an increase in all-cause mortality^[1,2]^.

Since we do not see the same amount of publications when it comes to PPM after mitral valve replacement (MVR), we decided to carry out a new systematic review with meta-analysis in order to evaluate the impact of PPM after MVR.

### Objectives

We aimed to investigate whether PPM increases the risk for death after MVR. This analysis was planned in accordance with current guidelines for performing comprehensive systematic reviews and meta-analysis, including the Preferred Reporting Items for Systematic Reviews and Meta-Analyses (PRISMA)^[3]^ guidelines. We pre-specified our analytical plan and registered the study protocol with PROSPERO, the international prospective register of systematic reviews (CRD42018089901).

## METHODS

### Eligibility Criteria

With the PICOS (Population, Intervention, Comparison, Outcome and Study design) strategy, studies were considered if: 1) the population comprised patients who underwent surgical MVR; 2) there was a group of patients who developed PPM (with an indexed effective orifice area (iEOA) - threshold of 1.20 cm^2^/m^2^) after MVR; 3) there was a control group of patients with no PPM; 4) outcomes included any of the following: perioperative or 10-year mortality rates as primary outcomes OR mean transprothetic gradient (mmHg), mean systolic pulmonary artery pressure (mmHg) and left ventricle ejection fraction (LVEF - %) as secondary outcomes; 5) studies were retrospective, prospective, randomized or non-randomized.

### Information Sources

The following databases were used (until December 2018): MEDLINE; EMBASE; CENTRAL/CCTR (Cochrane Controlled Trials Register); ClinicalTrials.gov; SciELO (Scientific Electronic Library Online); LILACS (Literatura Latino Americana em Ciências da Saúde); Google Scholar; and reference lists of relevant articles.

### Search

We conducted the search with the following terms: "mismatch OR PPM OR patient-prosthesis mismatch OR prosthesis-patient mismatch" and "MVR OR mitral valve replacement" OR "mitral valve prosthesis" OR "mitral valve implantation" OR "prosthetic mitral valve" OR "mitral prosthesis".

### Study Selection

The following steps were taken: 1) identification of titles of records through databases searching; 2) removal of duplicates; 3) screening and selection of abstracts; 4) assessment for eligibility through full-text articles; and 5) final inclusion in study. One reviewer followed steps 1 to 3. Two independent reviewers followed step 4 and selected studies. Inclusion or exclusion of studies was decided unanimously. When there was disagreement, a third reviewer made the final decision.

### Data Items

The crude endpoints were perioperative mortality, 10-year mortality, mean transprothetic gradient, mean systolic pulmonary artery pressure and LVEF.

### Data Collection Process

Two independent reviewers extracted the data. When there was disagreement about the data, a third reviewer checked them and made the final decision. From each study, we extracted patient characteristics, study design, and outcomes. When the data were not clearly available in the articles, we contacted the authors of the original articles by email.

### Summary Measures

The principal summary measures were *odds ratio* (OR) with 95% Confidence interval (CI) and *P*-values (considered statistically significant when *P*<0.05) for mortality and difference in means for the other outcomes. The meta-analysis was completed with the software Comprehensive Meta-Analysis (version 2, Biostat, Inc., Englewood, New Jersey).

### Synthesis of Results

Forest plots were generated for graphical presentations of clinical outcomes, and we performed the I^2^ test and χ^2^ test for the assessment of heterogeneity across the studies^[4]^. Inter-study heterogeneity was explored using the χ^2^ statistic, but the I^2^-value was calculated to quantify the degree of heterogeneity across the studies that could not be attributable to chance alone. When I^2^ was more than 50%, significant statistical heterogeneity was considered to be present. Each study was summarized by the OR or difference in means depending on the outcome, whose values were combined across the studies using a weighted DerSimonian-Laird random effects model^[5]^.

### Risk of Bias Across Studies

To assess publication bias, a funnel plot was generated for each outcome, statistically assessed by Begg and Mazumdar's test^[6]^ and Egger's test^[7]^.

### Sensitivity Analysis

We also investigated the influence of each study on the overall effect - by sequentially removing one study - in order to test the robustness of the main results, so that we could verify whether any study had an excessive influence on the overall results.

Furthermore, we analyzed the data as to the way the iEOA was measured (predicted from EOA measured in vitro by the manufacturer; or predicted from published normal reference values of EOA measured in vivo; or measured directly in each patient by Doppler-echocardiography following MVR).

### Meta-regression Analysis

Meta-regression analyses were performed to determine whether the effects of PPM on mortality were modulated by pre-specified factors. Meta-regression graphs describe the effect of PPM on the outcome (plotted as a log OR on the y-axis) as a function of a given factor (plotted as a mean or proportion of that factor on the x-axis). Meta-regression coefficients show the estimated increase in log OR per unit increase in the covariate. Since log OR > 0 corresponds to OR > 1 and log OR < 0 corresponds to OR < 1, a negative coefficient would indicate that as a given factor increases, the OR decreases.

The pre-determined modulating factors to be examined were male sex (%), female sex (%), age (years), hypertension (%), diabetes (%), renal failure (%), smoking (%), preoperative atrial fibrillation (%), bioprosthesis (%), mechanical valve (%), concomitant procedures (%), previous cardiac surgery (%) and LVEF (%).

## RESULTS

### Study Selection

**Fig. 1:**
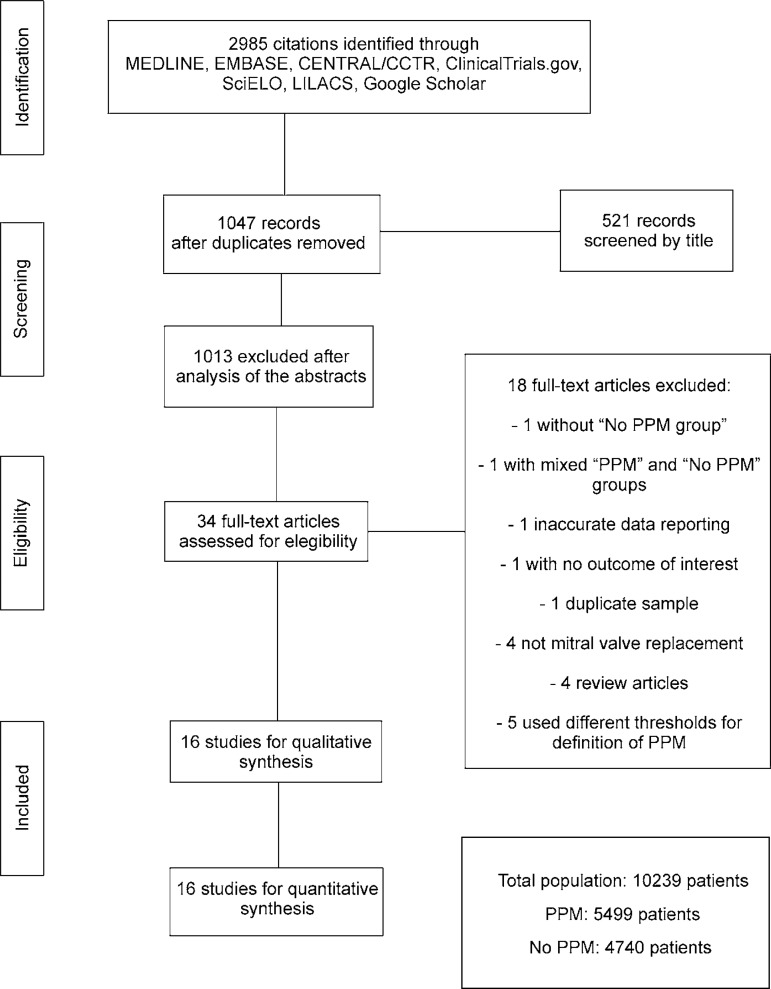
Flow diagram of studies included in data search. CCTR=Cochrane Controlled Trials Register; LILACS=Literatura Latino Americana em Ciências da Saúde; SciELO=Scientific Electronic Library Online

A total of 2,985 citations were identified, of which 34 studies were potentially relevant and retrieved as full-text. Sixteen publications^[8-23]^ fulfilled our eligibility criteria. Interobserver reliability of study relevance was excellent (Kappa=0.85). Agreement for decisions related to study validity was very good (Kappa=0.85). The search strategy can be seen in Figure 1.

### Study Characteristics

A total of 10,239 patients (with PPM: 5,499 patients; without PPM: 4,740 patients) were included from studies published from 2007 to 2018. The incidence of PPM after MVR was 53.7%, varying from 17.7% to 85.8%. The studies consisted of patients whose mean age ranged from 38.5 to 67.4 years. There were studies mostly from Asia, North America and Europe; only one from Latin America. All the publications were retrospective cohort studies, and 68.8% had some multivariate adjustment for possible confounders. Other characteristics are described elsewhere.

Synthesis of Results

**Fig. 2:**
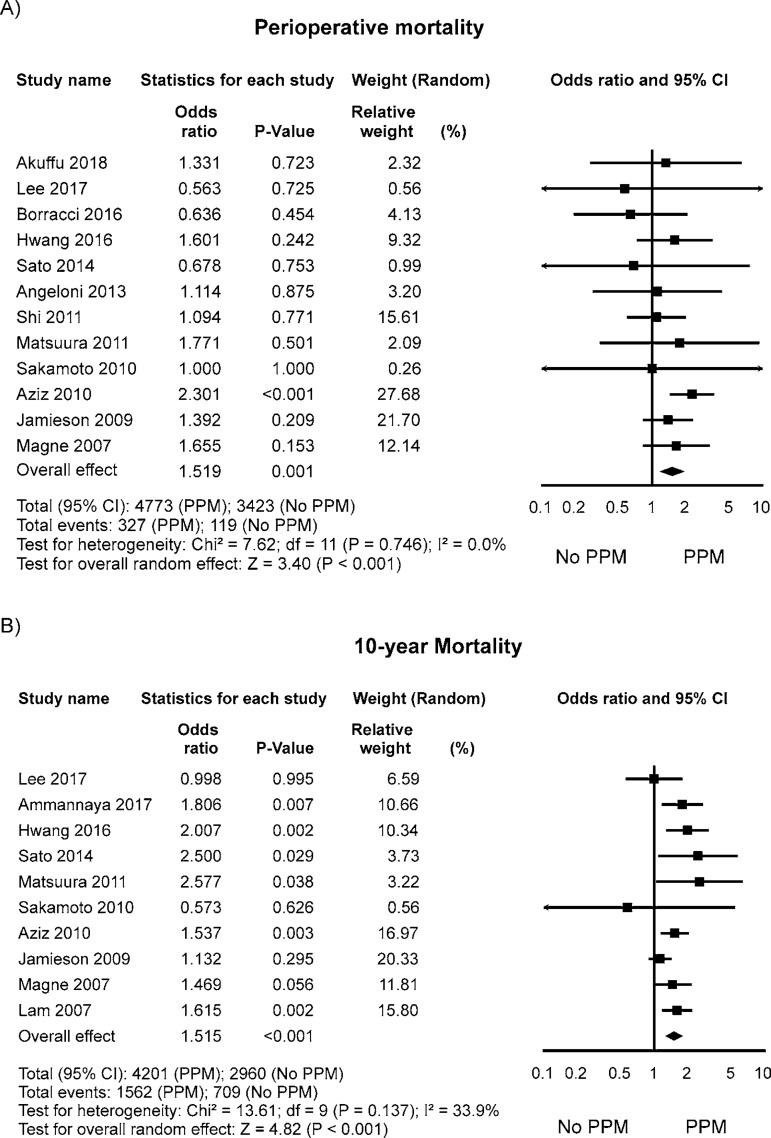
Odds ratio and conclusions plot of perioperative and 10-year mortality.This figure shows the summary effect of moderate/severe PPM on perioperative mortality. CI=confidence interval; PPM=patient-prosthesis mismatch

The OR for perioperative mortality in the PPM group compared with the no PPM group in each study is reported in Figure 2A. There was evidence of low statistical heterogeneity of treatment effect among the studies for perioperative mortality. The overall OR (95%CI) of perioperative mortality showed a statistically significant difference between the groups, with higher risk in the PPM group (random effect model: OR 1.519; 95%CI 1.194-1.931, *P*<0.001).

The OR for 10-year mortality in the PPM group compared with the no PPM group in each study is reported in Figure 2B. There was evidence of moderate statistical heterogeneity of treatment effect among the studies for 10-year mortality. The overall OR (95%CI) of 10-year mortality showed a statistically significant difference between the groups, with higher risk in the PPM group (random effect model: OR 1.515; 95%CI 1.280-1.795, *P*<0.001).

**Fig. 3:**
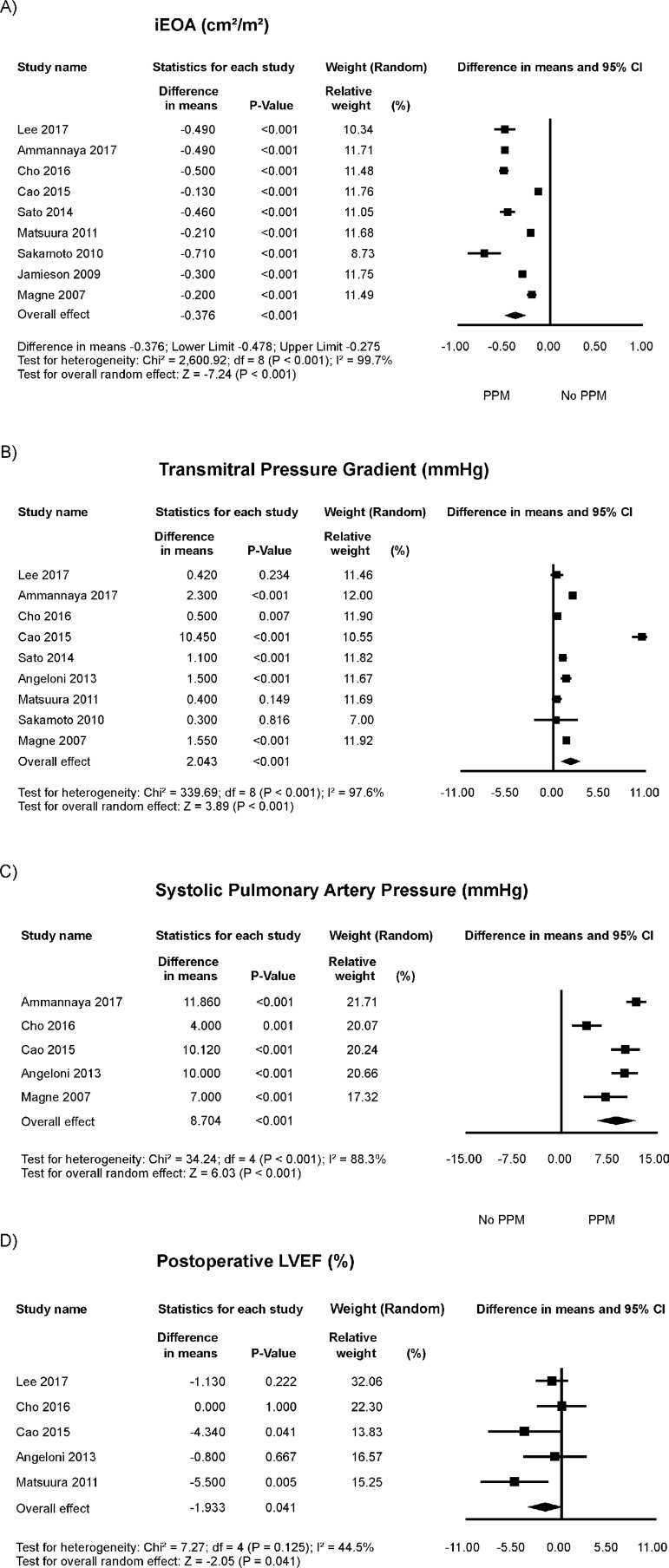
Odds ratio and conclusions plot of echocardiographic variables. This figure shows the summary difference in means for the outcomes. CI=confidence interval; iEOA=indexed effective orifice area; LVEF=left ventricle ejection fraction; PPM=patient-prosthesis mismatch

The differences in mean values of iEOA in PPM group compared with the no PPM group in each study are reported in Figure 3A. There was evidence for important heterogeneity of treatment effect among the studies for difference in means of iEOA. The overall difference in means was statistically significantly lower in the PPM group (random effect model: -0.376 cm^2^/m^2^; 95%CI -0.478 to -0.275; *P*<0.001).

The differences in mean values of transprosthetic gradient in PPM group compared with the no PPM group in each study are reported in Figure 3B. There was evidence for important heterogeneity of treatment effect among the studies for difference in means of transprosthetic gradient. The overall difference in means was statistically significantly higher in the PPM group (random effect model: 2.043 mmHg; 95%CI 1.015 to 3.072; *P*<0.001).

The differences in mean values of systolic pulmonary artery pressure in PPM group compared with the no PPM group in each study are reported in Figure 3C. There was evidence for important heterogeneity of treatment effect among the studies for difference in means of this outcome. The overall difference in means was statistically significantly higher in the PPM group (random effect model: 8.704 mmHg; 95%CI 5.877 to 11.531; *P*<0.001).

The differences in mean values of LVEF in PPM group compared with the no PPM group in each study are reported in Figure 3D. There was evidence for moderate heterogeneity of treatment effect among the studies for difference in means of LVEF. The overall difference in means was statistically significantly lower in the PPM group (random effect model: -1.933%; 95%CI -3.784 to -0.083; *P*=0.041).

Risk of Bias Across Studies

**Fig. 4:**
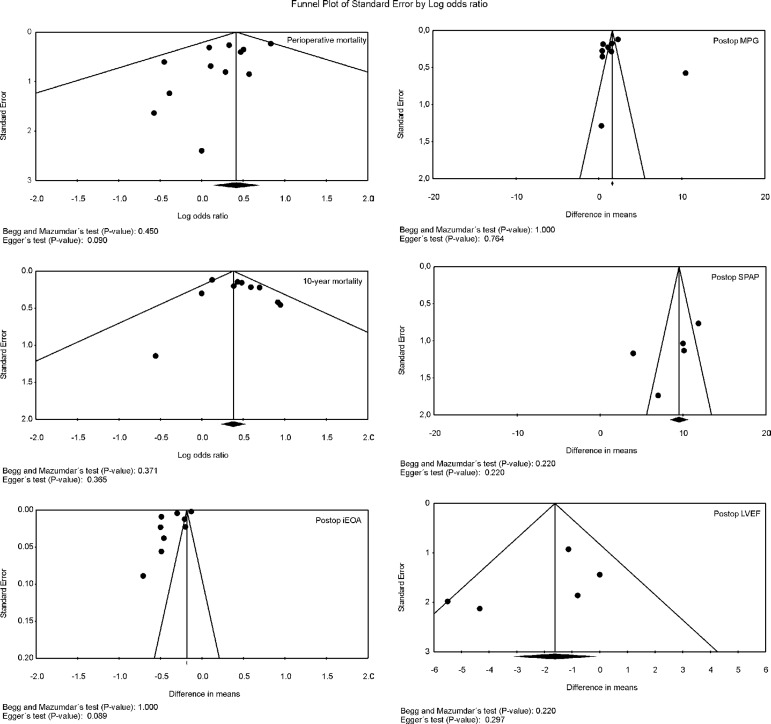
Publication bias. Funnel plot analysis of the outcomes perioperative and 10-year mortality.

Funnel plot analysis (Figure 4) disclosed no asymmetry around the axis for the outcomes, which means that we have low risk of publication bias related to these outcomes.

### Sensitivity Analysis

Sensitivity analyses performed by removing each single study from the meta-analysis (in order to determine the influence of individual data sets on the pooled ORs or difference in means) showed that none of the studies had a particular impact on the summary results.

**Fig. 5:**
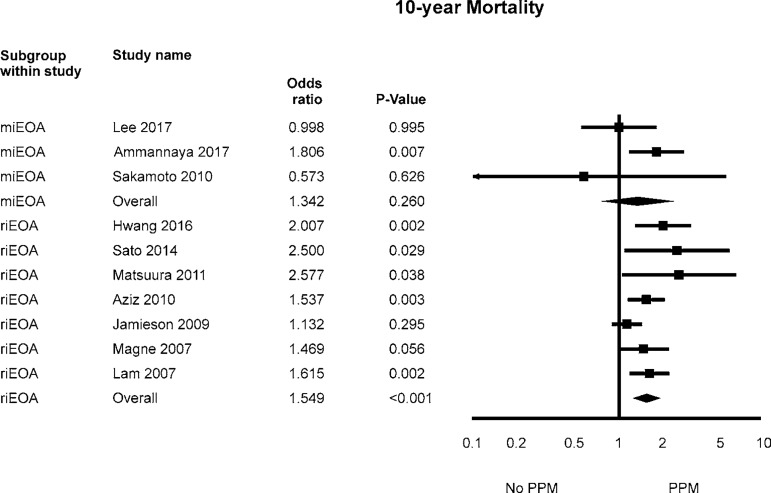
Sensitivity analysis. Funnel plot analysis of the outcomes for measured and referenced iEOA.

When we analyzed the data according to how the iEOA was measured in order to define the presence of PPM (whether *in vivo, in vitro* or measured by echocardiography), we found that the overall OR (95%CI) for 10-year mortality showed a statistically significant difference with higher risk in the "PPM" group only when the iEOA was measured by the referenced iEOA (Figure 5).

### Meta-Regression Analysis

None of the pre-determined factors (sex, age, hypertension, diabetes, renal failure, smoking, preoperative atrial fibrillation, type of valve, concomitant procedures, previous cardiac surgery, LVEF) showed any particular modulating effect on the results.

## DISCUSSION

### Summary of Evidence

To the best of our knowledge, this is the largest meta-analysis of studies performed to date that provides additional value by demonstrating that patients with PPM have higher risk for mortality in comparison to those with no PPM after MVR. We also observed that more than half of the patients leave the operation room with significant PPM, having this aspect a negative impact not only on the long term, but also already on the perioperative period. Patients with PPM also showed higher systolic pulmonary artery pressure and transprosthethic gradient.

### Clinical Implications According to Geographical Localization

These results have important clinical implications given that PPM is a potentially modifiable risk factor. Considering that we observed an incidence of PPM after MVR higher than 50% that implies higher rates of mortality, it would be no exaggeration to say that this problem has reached epidemic proportions and surgeons would have to take measures to counter the risk of PPM after MVR in order to decrease mortality.

We must highlight that not all the countries of the world can afford the so-called "new generation" prostheses with larger effective orifice areas. In the largest country of Latin America, Brazil, for example, more than 90% of the patients are operated on at centers of the public health system, where the patients cannot receive these new generation prostheses simply because they are not available in the system due to their prices. Indeed, these new models of prostheses are easily available in Europe and in the USA, but not within the public health systems in Latin America (including Brazil), Africa and most part of Asia, where surgeons have to work with other types of prostheses, with "older" technology. Moreover, in Europe and in the USA, PPM is often diagnosed in small, elderly women whereas in developing countries, surgeons mostly come across younger patients who are part of the working age population who suffer from rheumatic heart disease.

### What might be the solution to this problem?

Although several procedures have been described for enlargement of the aortic anulus, such as the Nicks, Manougian, and Konno procedures, there are very few options for enlargement of the mitral anulus. This is because of the presence of the circumflex coronary artery, membranous ventricular septum, the conduction bundle and the aortic valve, which encircle the mitral anulus. Some surgeons have performed supra-annular mitral valve replacement when the mitral anulus has been inadequate to accept an adequate-sized mitral prosthesis. Supra-annular mitral valve replacement involves insertion of the prosthesis entirely within the left atrium, thereby creating a ventricularized portion of the left atrium. Early results have been discouraging, with high mortality and the risk of left atrial diastolic dysfunction and pulmonary vein stenosis^[24-26]^, although other reports have shown good results^[27]^. Furthermore, supra-annular MVR does not protect against leaflet entrapment or valve thrombosis, which occurred in 13% of the patients with supra-annular MVR reported by Kanter et al.^[27]^.

Jonas et al.^[28]^ described a surgical technique used in the context of pediatric cardiac surgery that might well be applied in the context of adult cardiac surgery in order to enlarge the mitral annulus. Myers et al.^[29]^ studied 205 mitral valve replacement procedures carried out between 1990 and 2012, and mitral annulus enlargement techniques were analyzed, which included intraoperative balloon dilation of the annulus under direct vision, radial annular incisions, and patch augmentation of the aorto-mitral continuity. By using these techniques, it was possible to enlarge the annulus. However, the authors also underscored that there is a nontrivial risk of heart block with annulus upsizing, which deserves further study.

### Sources of Heterogeneity

The statistical heterogeneity in the analyses of the continuous variables might be related to various sources - for example, to the type of prosthesis (bioprosthetic or mechanical valve). The type of valve could be a confounding factor, as mechanical valves are implanted more often in younger patients, who generally have a more active lifestyle and faster metabolism. Just a word of caution: most of the studies were composed of a mixed pool of patients (receiving biological or mechanical valves) and we were not able to break down the data in those studies, otherwise we could have gone deeper in the analysis.

Another important source of heterogeneity might be the definition of PPM applied in the studies. Indeed, when we carried out subgroup analyses according to the method used to define PPM, we observed that the use of predicted (measured in vitro by the manufacturers or *in vivo* from published normal reference values) or measured iEOA had different impacts on the pooled results for 10-year mortality rates.

### Risk of Bias and Limitations of the Present Study

There are inherent limitations with meta-analyses, including the use of cumulative data from summary estimates. Patient data were gathered from published data, not from individual patient follow-up. Access to individual patient data would have enabled us to conduct further subgroup analysis and propensity analysis to account for differences between the treatment groups. This meta-analysis included data from studies that reflect the "real world" but, on the other hand, are less limited by publication bias, treatment bias, confounders, and a certain tendency to overestimate treatment effects observed in the observational studies, since patient selection alters outcome and, thus, makes non-randomized studies less robust.

Moreover, considerable statitiscal heterogeneity was observed in some analyses, but we used the random-effects model to counterbalance this aspect. We also observed low risk of publication bias in the outcomes. We must remind the readers of the fact that research with statistically significant results is more likely to be submitted to medical journals and published than work with null or non-significant results, being the former also more likely to appear more prominently in English, in higher impact journals. All of the aforementioned aspects lead to the appearance of publication biases, but, in this case, we cannot state that the impact of PPM on mortality rates observed in our study is solely due to bias.

## CONCLUSION

This meta-analysis found that PPM is associated with a significant increase in perioperative and long-term mortality rates after surgical MVR. Hence, a particular effort should be made to prevent PPM, and especially, severe PPM, at the time of MVR.

**Table:** 

Authors' roles & responsibilities	
MPBOS	Substantial contributions to the conception or design of the work; or the acquisition, analysis, or interpretation of data for the work; agreement to be accountable for all aspects of the work in ensuring that questions related to the accuracy or integrity of any part of the work are appropriately investigated and resolved; final approval of the version to be published
LRPC	Substantial contributions to the conception or design of the work; or the acquisition, analysis, or interpretation of data for the work; final approval of the version to be published
SCR	Substantial contributions to the conception or design of the work; or the acquisition, analysis, or interpretation of data for the work; final approval of the version to be published
AMM	Substantial contributions to the conception or design of the work; or the acquisition, analysis, or interpretation of data for the work; final approval of the version to be published
PP	Drafting the work or revising it critically for important intellectual content; final approval of the version to be published
MAC	Drafting the work or revising it critically for important intellectual content; final approval of the version to be published
RCL	Drafting the work or revising it critically for important intellectual content; final approval of the version to be published
